# Prognostic impact of detecting viable circulating tumour cells in gastric cancer patients using a telomerase-specific viral agent: a prospective study

**DOI:** 10.1186/1471-2407-12-346

**Published:** 2012-08-09

**Authors:** Hiroaki Ito, Haruhiro Inoue, Norimasa Sando, Satoshi Kimura, Keigo Gohda, Jun Sato, Katsuhiro Murakami, Shun Ito, Noriko Odaka, Hitoshi Satodate, Shin-ei Kudo

**Affiliations:** 1 Digestive Disease Center, Showa University Northern Yokohama Hospital, 35-1 Chigasakichuo, Tsuzuki-ku, Yokohama 224-8503, Japan; 2 Department of Laboratory Medicine and Central Clinical Laboratory, Showa University Northern Yokohama Hospital, 35-1 Chigasakichuo, Tsuzuki-ku, Yokohama, 224-8503, Japan; 3 Central Research Laboratories, Sysmex Corporation, 4-4-4 Takatsukadai, Nishi-ku, Kobe, 651-2271, Japan

**Keywords:** Circulating tumour cells, Gastric cancer, Telomerase

## Abstract

**Background:**

The identification of circulating tumour cells (CTCs) in peripheral blood is a useful approach to estimate prognosis, monitor disease progression, and measure treatment effects in various malignancies. However, clinical relevance of CTCs is controversial. We attempted to detect viable CTCs in the peripheral blood of gastric cancer patients using a telomerase-specific viral agent.

**Methods:**

We took a 7.5-ml blood sample from 65 treatment-negative gastric cancer patients before surgery and 10 healthy volunteers. We detected viable CTCs in the blood samples after incubating them with a telomerase-specific, replication-selective, oncolytic adenoviral agent carrying the green fluorescent protein (GFP) gene (OBP-401). GFP-positive CTCs were defined as having a diameter of at least 7.735 μm; this threshold was determined by receiver operating characteristic curve analysis. GFP-positive cells were counted under a fluorescence microscope.

**Results:**

There was a significant difference in overall survival among the patients with 0–4 and those with ≥5 GFP-positive CTCs in the stage I–IV disease group and stage II–IV advanced disease group. The number of GFP-positive CTCs was not related to cancer stage. Among the pathological findings, the number of GFP-positive CTCs was only significantly related to venous invasion, although there were trends towards more GFP-positive CTCs with disease progression (tumour depth, lymph node metastasis, distant metastasis, lymphatic invasion, and histological type).

**Conclusions:**

There was a significant relationship between the number of GFP-positive CTCs and overall survival in the patients with gastric cancer. The detection of CTCs using OBP-401 may be useful for prognostic evaluation.

**Trial registration:**

University Hospital Medical Information Network in Japan, UMIN000002018.

## Background

Distant metastasis of a solid tumour is a strong prognostic factor [1-3]. The existence of circulating tumour cells (CTCs) in peripheral blood suggests that a patient is in a systemic disease phase [4]. The identification of CTCs in peripheral blood is a useful approach to estimate prognosis, monitor disease progression, and measure treatment effects in breast, prostate, skin, colon and gastrointestinal malignancies. Therefore, various methods have been developed to detect CTCs, and are occasionally used in combination. Common techniques for the enrichment and detection of CTCs are density gradient separation [5], direct enrichment by filtration [6,7], immunomagnetic separation [8], flow cytometry [9], real-time reverse transcriptase polymerase chain reaction (RT-PCR) [10,11], and microchip technology [12].

Cell enrichment by density gradient separation is performed using commercial kits such as OncoQuick® (Greiner, Frickenhausen, Germany) [13] and Lymphoprep® (Nycomed, Oslo, Norway) [14]. Density gradient separation is based on the theory that different types of cells can be separated according to their density. Therefore, it is difficult to extract all CTCs because of cell migration. RT-PCR is one of the most common methods of tumour cell detection because of its high sensitivity and specificity, assuming adequate primer and probe design. However, false-positive results may occur because of its technical delicacy and high sensitivity [15,16].

Immunomagnetic cell enrichment, such as that performed by the CellSearch System® (Veridex, LLC, Raritan, NJ, USA) [17], is currently the most commonly used technique to enrich and detect CTCs [18-21]. The advantage of immunomagnetic cell separation is that CTCs can be visualised with a fluorescent microscope. Cells detected with antibodies against epithelial markers (epithelial cell adhesion molecules; EpCAMs) are determined to be CTCs. Therefore, this technique can provide false-positive results based on normal epithelial marker expression by non-tumour cells, and false-negative results can arise based on the lack of selective marker expression on tumour cells. As a result of the limitations associated with the above approaches, a new technique is needed to detect viable CTCs precisely.

Telomerase plays important roles in carcinogenesis, cancer invasion, and metastasis [22-24]. We have developed a technique to exploit high telomerase activity in cells. This technique uses a telomerase-specific, replication-selective modified viral agent (OBP-401; TelomeScan®, Oncolys BioPharma, Tokyo, Japan) in which the human telomerase reverse transcriptase (*TERT*) gene promoter is inserted into the E1 region, and the green fluorescent protein (GFP) gene is placed under the control of the cytomegalovirus promoter in the E3 region as a marker of viral replication [25]. It has been reported that OBP-401 can be used to detect viable CTCs among normal blood cells [26,27].

Here, we applied the assay to detect viable CTCs with the potential for metastasis in gastric cancer patients. We detected GFP-positive CTCs. In contrast, it is possible that non-cancer cells emit GFP fluorescence after OBP-401 infection [28]. Therefore, we selected cells larger than a threshold determined by comparison between healthy volunteers and patients, because CTCs are larger than normal blood cells [7,29,30]. We studied the association of GFP-positive CTC number with survival and pathological indices of disease progression.

## Methods

### Patients and healthy volunteers

The patients included in this preliminary study were those undergoing planned surgical treatment; patients who were suitable for endoscopic mucosal resection or endoscopic submucosal dissection were excluded. The inclusion criteria were: (i) histologically proven adenocarcinoma of the stomach by endoscopic biopsy; (ii) clinical solitary tumour; (iii) no prior endoscopic resection, chemotherapy, or radiotherapy; (iv) age 20–80 years; (v) Eastern Cooperative Oncology Group performance status [31] 0 or 1; (vi) sufficient organ function; and (vii) written informed consent. The exclusion criteria were: (i) synchronous or metachronous malignancy; (ii) pregnant or breastfeeding women; (iii) active or chronic viral hepatitis; (iv) active bacterial or fungal infection; (v) diabetes mellitus; (vi) systemic administration of corticosteroids; and (vii) unstable hypertension. The disease stage in the patients was pathologically characterised using the seventh edition of the TNM classification of the International Union Against Cancer [32]. The depth of the tumour in three patients without gastrectomy and the regional lymph node status of five patients without sufficient lymphadenectomy were diagnosed surgically.

All the patients attended our hospital regularly after surgery, and were checked every 3 months. The patients also underwent endoscopy and computed tomography at least once a year, according to their disease stage and course.

We also recruited healthy volunteers to act as controls. All healthy volunteers were employees of Sysmex Corporation and included seven men (mean age 31.4 years, range 24–39 years) and three women (mean age 33.7 years, range 26–48 years). All volunteers underwent medical check-ups upon employment and annually; check-ups included medical interviews, auscultation, chest radiographs, and blood and urine analyses. Furthermore, we performed individual interviews before sample collection; any volunteer who was currently receiving medical treatment, pregnant or breastfeeding, or who had donated blood within the past month was excluded.

### Virus

OBP-401, a telomerase-specific, replication-selective adenoviral agent in which the *TERT* promoter element drives the expression of the *EIA* and *EIB* genes, and into which the GFP gene is integrated, was used in this study. Sensitivity and specificity of the assay using OBP-401 have been studied previously by Kim et al. [27]. The test was repeated five times test in the sample including 1 MDA-MB-468 (breast carcinoma) cells and 7.5 ml blood, and the numbers of GFP-positive cells were 1, 1, 1, 2 and 3; in the sample including 20 MDA-MB-468 (breast carcinoma) cells, the numbers of GFP-positive cells were 15, 17, 19, 22 and 24. Viral samples were stored at −80°C.

### Cell lines and culture

The A549 (lung carcinoma), HepG2 (hepatocellular carcinoma), HEC-1 (endometrial adenocarcinoma), KATO-III (gastric carcinoma), and SBC-3 (small cell lung carcinoma) cell lines were obtained from the Health Science Research Resources Bank (Osaka, Japan). The LNCaP (prostate adenocarcinoma) and OVCAR-3 (ovarian carcinoma) cell lines were obtained from the Riken Cell Bank (Tokyo, Japan). The cells were cultured according to the vendor’s specifications. MDA-MB-468 cells were cultured as previously described [27].

### Sample preparation and cell counting

A 7.5-ml peripheral vein blood sample was obtained from each patient before surgery and from each volunteer. The samples were drawn into tubes containing citric acid, phosphoric acid, and dextrose, and stored at 4°C. The assay was started within 48 h.

The samples were centrifuged for 5 min at 540 g and the plasma phase was removed. The cells were then washed four times with phosphate-buffered saline (PBS) and twice with Roswell Park Memorial Institute medium. The samples were infected with 4 × 10^8^ plaque-forming units (PFU) of OBP-401 viruses by incubation in the medium for 24 hours at 37°C.

After dead cell staining by the red-fluorescent reactive dye L23102 (Life Technologies Corporation, Carlsbad, CA, USA), OBP-401 viruses were inactivated and cells were fixed with 2% paraformaldehyde (PFA) for 20 min at room temperature.

**Figure 1:**
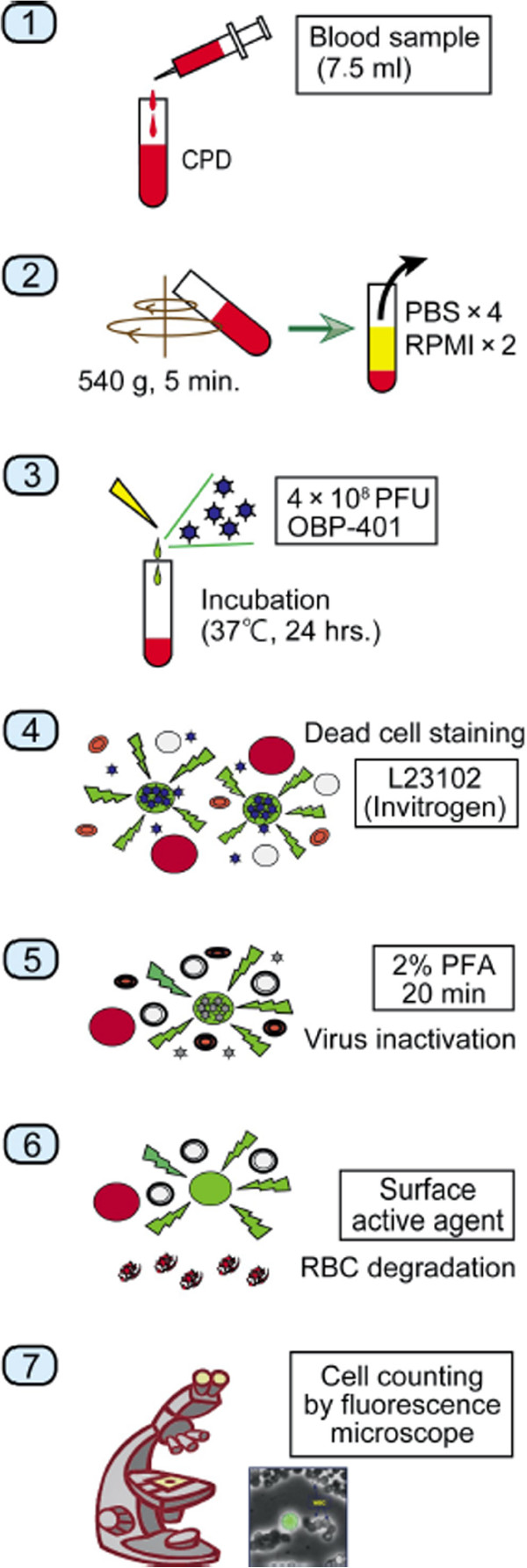
**Protocol and procedure of the “GFP-CTC Assay”.** Each 7.5-ml peripheral vein blood sample was obtained from patients before surgery and from volunteers. The samples were drawn into tubes containing citric acid, phosphoric acid and dextrose. The samples were centrifuged and the plasma phase was removed. After cell washing using PBS and Roswell Park Memorial Institute medium, the samples were infected with 4 × 10^8^ PFU of OBP-401 virus. OBP-401 virus was inactivated and cells were fixed with 2% paraformaldehyde (PFA). The samples were treated with a surface-active agent to degrade red and white blood cells. Two glass slide samples from 7.5 ml blood were prepared for microscopic analysis. All GFP-positive cells on the two slides were counted using a computer-controlled fluorescent microscope.

The samples were treated with a surface-active agent (Emalgen 2025 G; Kao Chemicals, Tokyo, Japan) for 10 min at 40°C to degrade red blood cells. Finally, 7.5 ml blood was used to create two glass slide samples for microscopic analysis. All GFP-positive cells on the two slides were counted, using a computer-controlled fluorescent microscope (IX71; Olympus, Tokyo, Japan) and an examiner blinded to the sample details. The total assay time was 27.5 h (Figure 1).

### Determination of GFP fluorescence intensity threshold

**Figure 2:**
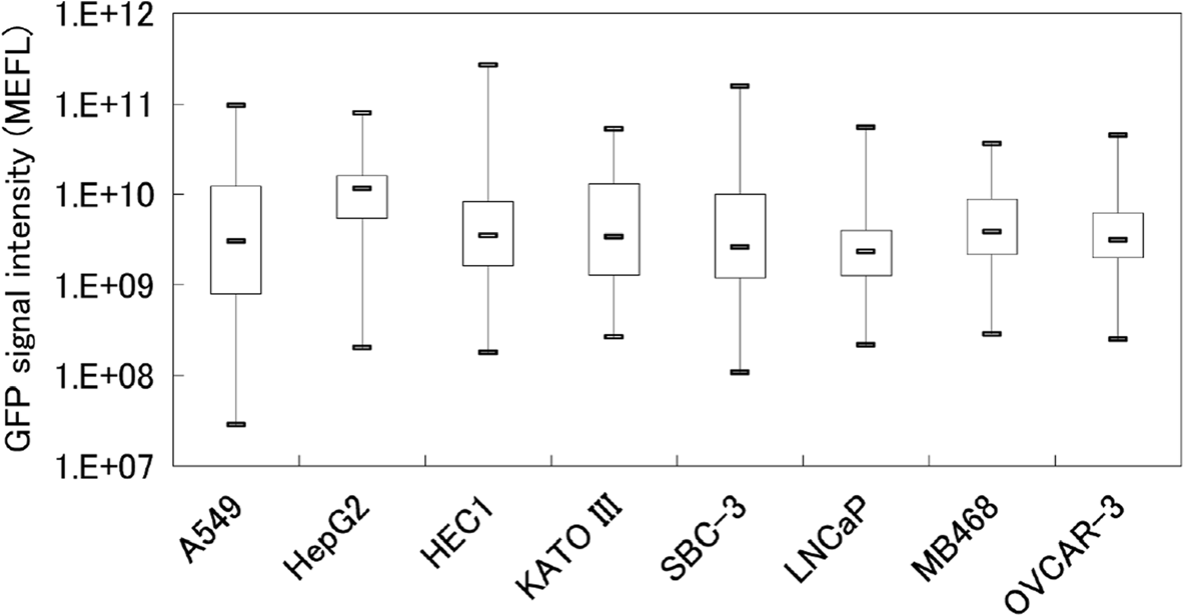
**GFP signal intensities from various cancer cell lines.** The bottom and top of the box are the lower and upper quartiles, and the band of the box is the median. The lines on the end of the whiskers are minimum and maximum. The y axis represents GFP signal intensity on a log scale.

The threshold for GFP fluorescence intensity was determined as follows. Approximately 30,000 cultured cells were spiked into 7.5-ml blood samples from healthy volunteers, which were spiked with various cancer cell lines: A549, HepG2, HEC-1, KATO-III, SBC-3, LNCaP, MDA-MB-MB468 and OVCAR-3. Blood samples were subjected to CTC detection assay, and then detectable cells were counted by fluorescence microscopy. More than 100 cells were analysed in each sample. We determined 2.85 × 10^7^ mean equivalent fluorochrome to be the threshold for GFP signal intensity from the minimal GFP intensity level observed in the blood samples spiked with the cell lines (Figure 2). Data are reported as the numbers of GFP-positive CTCs per 7.5-ml peripheral blood sample.

### Immunostaining

Phycoerythrin-labelled anti-human CD45 antibody (BioLegend, San Diego, CA, USA) was diluted 1:5 and Pacific Blue-labelled anti-human CD326 (EpCAM) antibody (BioLegend) was diluted 1:10 in PBS containing 2% foetal bovine serum. Cells were incubated with diluted antibodies for 30 min at 25°C. After washing with PBS containing 2% foetal bovine serum, cells were mounted on glass slides and analysed using a fluorescence microscope (IX71; Olympus).

### Statistical analysis

Statistical analysis was performed using JMP Statistical Discovery 9.0.2 (SAS Institute, Cary, NC, USA). To determine the cell diameter threshold, the detected GFP-positive CTCs were analysed using a receiver operating characteristic (ROC) curve between the samples from gastric cancer patients and those from volunteers. For multiple group comparisons, homogeneity of variance was assessed by the Levene test. For parametric comparisons we used analysis of variance, and for non-parametric comparisons we used the Wilcoxon and the Kruskal–Wallis tests. We used Fisher’s exact and *χ*^2^ tests with a 2 × 2 and a 4 × 2 table, respectively, to compare the clinicopathological characters in the patient group. Kaplan–Meier curves of estimated disease-free survival and overall survival were generated, and comparisons among the groups were performed using a two-sided log-rank test. *P* values ≤0.05 were considered statistically significant.

The study was approved by the Institutional Review Board of the Showa University, Northern Yokohama Hospital. We explained the study protocol to the patients and volunteers before they gave written informed consent. This study was registered with the University Hospital Medical Information Network in Japan, number 000002018.

## Results

### Participant characteristics

Sixty-five patients with gastric adenocarcinoma (46 men and 19 women; mean age 58.8 years, range 33–76 years) who underwent surgery at the Digestive Disease Center of the Showa University Northern Yokohama Hospital between September 2009 and May 2011 were included in this study. Twenty-nine had distal gastrectomy, 32 had total gastrectomy, and four had exploratory laparotomy. The patients’ characteristics are summarised in Table 1. Twenty-eight of the 65 patients received chemotherapy after surgery. The control group comprised 10 healthy volunteers.

**Table 1:** Patient characteristics and pathological findings

**Parameters**	**Numbers of patients**
Sex	
Male	46
Female	19
Age (mean, range)	58.8, 33-76
Gastrectomy	
Distal	29
Total	32
None	4
Surgical approach	
Laparoscopy	54
Open laparotomy	11
Curability	
R0	57
R1	0
R2	8
TNM Stage	
I	40
II	6
III	10
IV	9
Tumour depth of invasion	
T1	36
T2	8
T3	9
T4	12
Lymph node metastasis	
N0	39
N1	5
N2	6
N3	15
Distant metastasis	
M0	56
M1	9
Main histological type†	
Differentiated	25
Undifferentiated	40
Lymphatic invasion	
LX	4
L0	35
L1	26
Venous invasion	
VX	4
V0	35
V1-2	26

† Well or moderately differentiated adenocarcinoma and papillary adenocarcinoma were categorized as differentiated type. Signet-ring cell carcinoma, poorly differentiated adenocarcinoma and mucinous adenocarcinoma were categorized as undifferentiated type.

### GFP-positive CTCs in peripheral blood

Using a fluorescence microscope, cells with fluorescent emissions ≥2.85 × 10^7^ mean equivalent fluorochrome were counted as GFP-positive cells.

**Figure 3:**
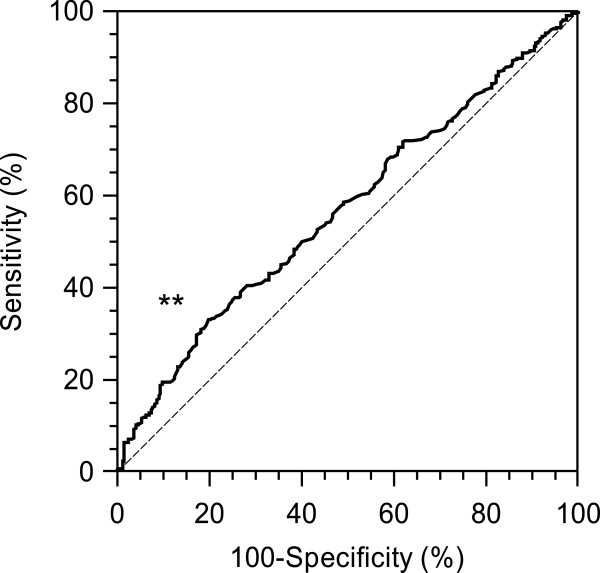
**Comparison of cell diameter between patients and volunteers.** To determine the threshold, we compared the diameters of cells from gastric cancer patients and controls by ROC analysis. There was a significant difference between the gastric cancer patients and the controls (*p* < 0.001; area under the curve = 0.57; 95% confidence interval = 0.54–0.60).

Various sizes of GFP-positive cells were observed in each sample; therefore, it was difficult to identify one representative cell among the GFP-positive cells for comparison between patients and healthy volunteers. Therefore, to avoid using an arbitrary value, we decided to use the optimum threshold derived from the ROC analysis based on the cell size, that is, 7.735 μm, as the threshold to define GFP-positive CTCs (Figure 3).

**Figure 4:**
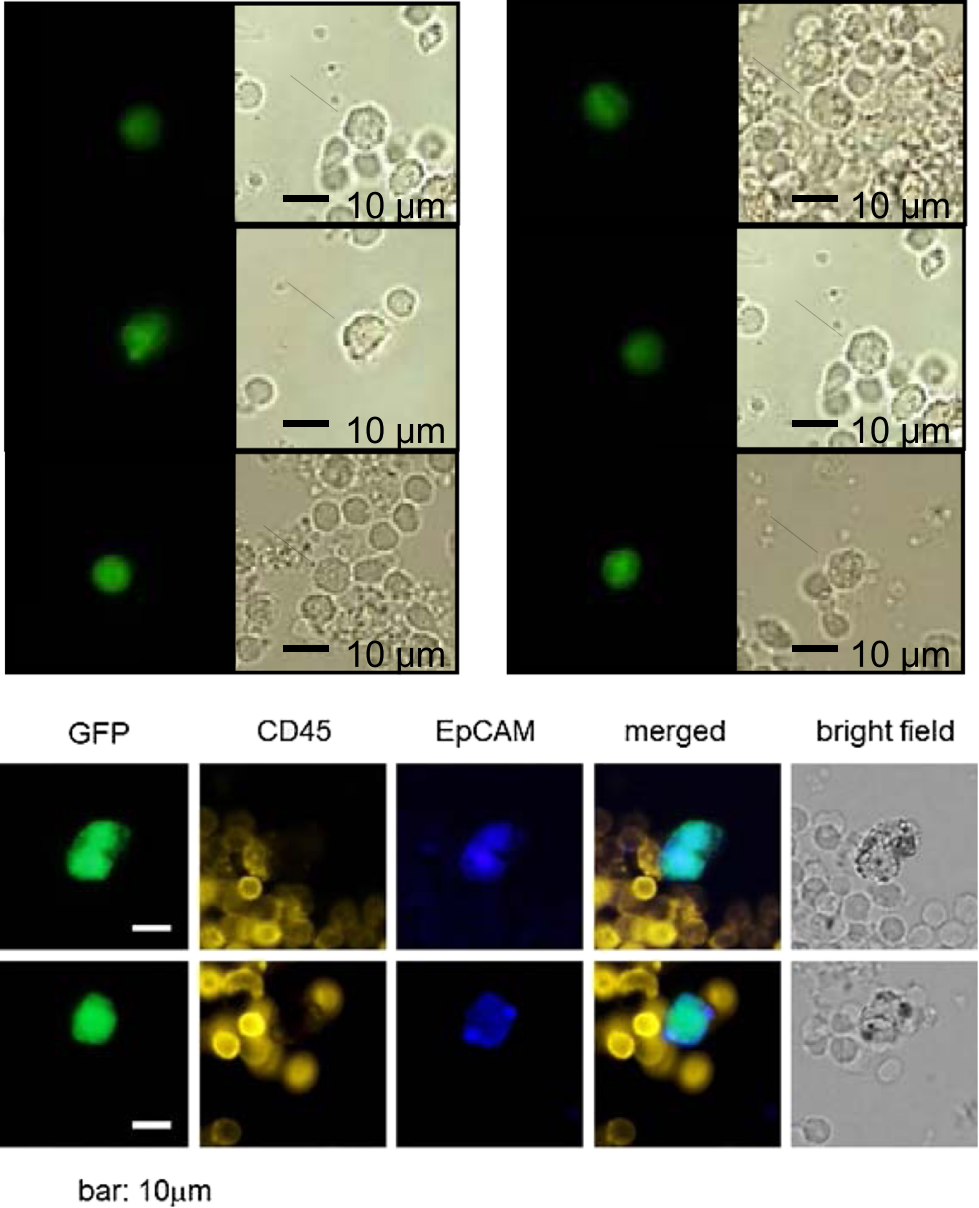
**Examples of microscopic images.** Representative images from two gastric cancer samples of GFP-positive CTCs and immunocytochemical analysis of anti-EpCAM- and anti-CD45-positive cells. Cells were counted using a computer-controlled fluorescence microscope by an examiner blinded to the sample status. Scale bar, 10 μm.

By immunohistochemical staining using anti-EpCAM and anti-CD45 antibodies, we confirmed that GFP-positive CTCs were EpCAM-positive and CD45-negative (Figure 4).

**Figure 5:**
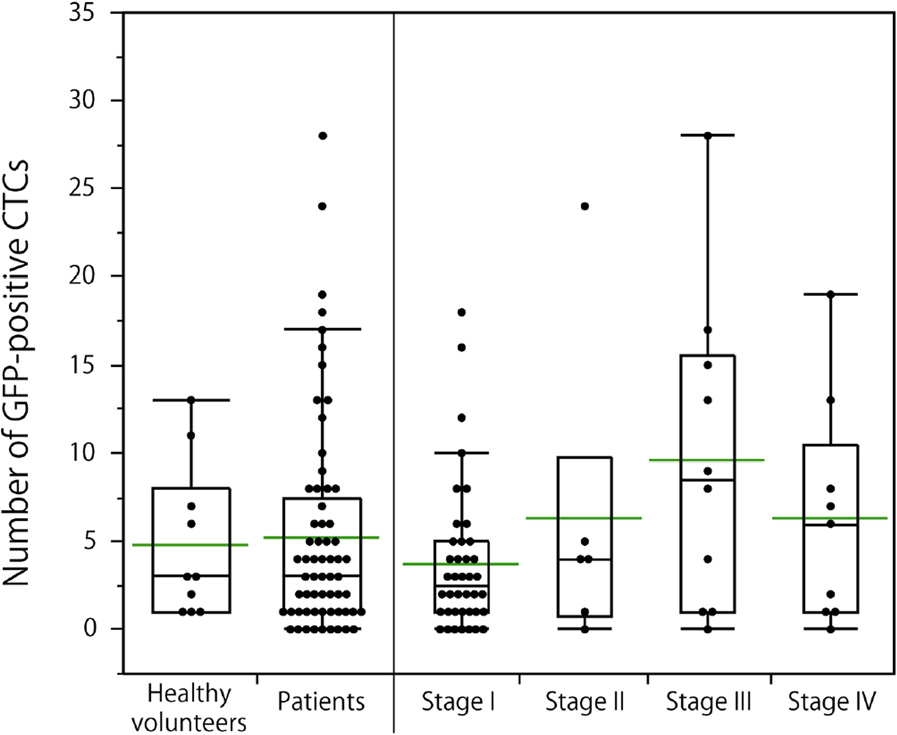
**Number of GFP-positive cells.** The dots are the numbers of GFP-positive CTCs in the patient samples. The bottom and top of the box represent the lower and upper quartiles, and the band across the box shows the median. The lower and upper bars at the ends of the whiskers show the lowest data point within 1.5 interquartile ranges of the lower quartile, and the highest data point within 1.5 interquartile ranges of the upper quartile, respectively. The green bars indicate mean value.

The numbers of GFP-positive CTCs in the peripheral blood samples are shown in Figure 5. There was no significant difference among the detection rates of GFP-positive CTCs in the samples representing each cancer stage.

### Association of GFP-positive CTCs with survival

The mean number of GFP-positive CTCs in the samples from 10 healthy volunteers was 4.8. We divided the patients into two groups based on the numbers of GFP-positive CTCs: 0–4 and ≥5. The clinicopathological characteristics of the two groups are summarised in Table 2. There was no significant difference between the two groups except that there was only one category of lymphatic metastasis.

**Table 2:** Clinicopathological characters of two patient groups by number of CTCs

**Number of CTCs**	**0 - 4**	**5 ≤**	***P *value**
**Number of subjects**	**41**	**24**	
TNM: Stage			0.1586
Stage I	29	11	
Stage II	4	2	
Stage III	4	6	
Stage IV	4	5	
TNM: T category			0.2753
T1	26	10	
T2	5	3	
T3	5	4	
T4	5	7	
TNM: N category			0.0257 *
N0	27	12	
N1	5	0	
N2	4	2	
N3	5	10	
TNM: M category			0.2121
M0	37	19	
M1	4	5	
Main histological type			0.6844
Differentiated type	15	10	
Undifferentiated type	26	14	
Lymphatic invasion			0.3177
Lx	2	2	
L0	25	10	
L1	14	12	
Venous invasion			0.1293
Vx	2	2	
V0	26	9	
V1-2	13	13	
Surgery			0.2124
Curative resection	38	19	
Non-curative resection	1	3	
No resection	2	2	
Postoperative chemotherapy			0.1672
Presence	15	13	
Absence	26	11	

* * Significant difference.

**Figure 6:**
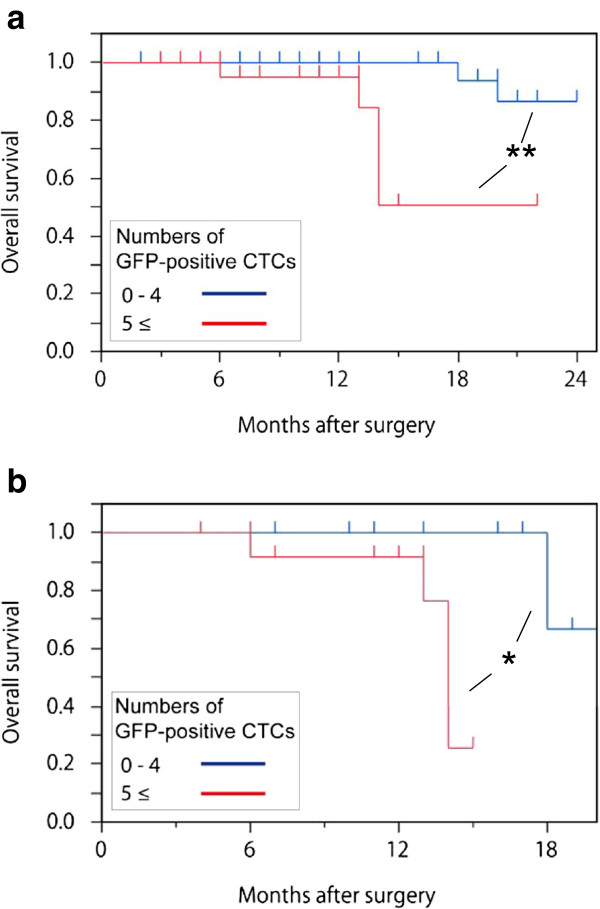
**Overall survival.** (**a**) Overall survival of all patients. (**b**) Overall survival of patients with stage II–IV disease. Survival was compared according to the number of CTCs using Kaplan–Meier analysis and the log-rank statistics. **p < 0.01, * *p* < 0.05.

Figure 6a shows the Kaplan–Meier curves for overall survival in the two groups: the patients with 0–4 CTCs and those with ≥5. There was a significant difference between the two groups (*p* = 0.0021). Furthermore, in the advanced gastric cancer patients with stage II–IV disease, there was a significant difference between the two groups (*p* = 0.0126) (Figure 6b).

### Association of GFP-positive CTCs with pathological indices

**Figure 7:**
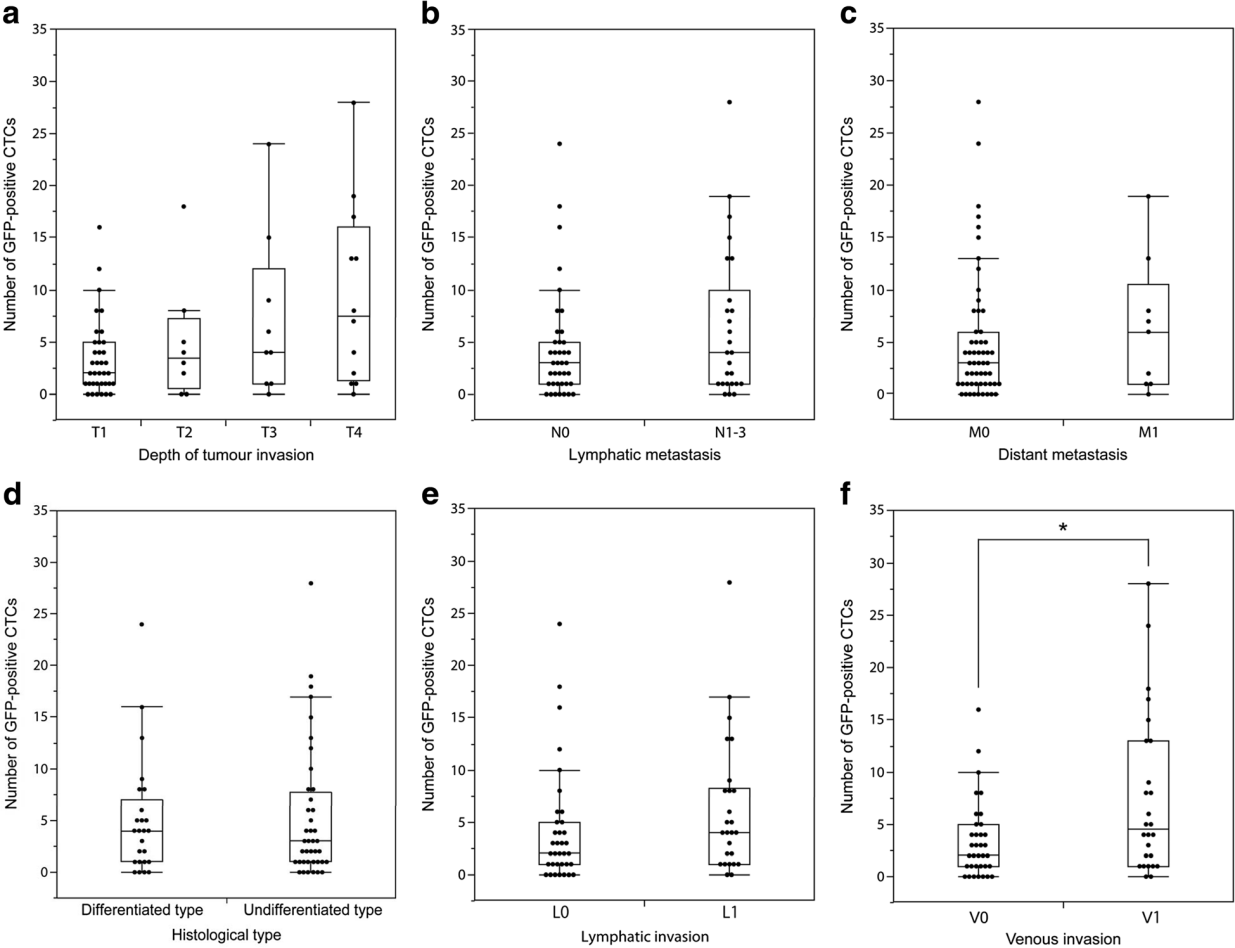
**Relationship between number of GFP-positive CTCs in a 7.5-ml blood sample from gastric cancer patients and pathological findings in the patients.** The dots are the numbers of GFP-positive CTCs in the patient samples. The bottom and top of the box represent the lower and upper quartiles, and the band across the box shows the median. The lower and upper bars at the ends of the whiskers show the lowest data point within 1.5 interquartile ranges of the lower quartile, and the highest data point within 1.5 interquartile ranges of the upper quartile, respectively. **a**, Tumour invasion depth (T1–T4 indicate increasing depth). **b**, Lymphatic metastasis (N0 = negative, N1–3 = positive). **c**, Distant metastasis (M0 = negative, M1 = positive). **d**, Histological type (differentiated type and undifferentiated type). **e**, Lymphatic invasion (L0 = negative, L1 = positive). **f**, Venous invasion (V0 = negative, V1 = positive), *p < 0.05. Although only venous invasion displayed a statistically significant difference, there were some trends towards an increase in CTC number with increasing disease progression.

There was no significant relationship between the number of GFP-positive CTCs and cancer stage (*p* = 0.2313) (Figure 5). Although no statistical significance was observed, the number of GFP-positive CTCs tended to increase with the progression of the primary tumour (*p* = 0.1521) (Figure 7a). The number of CTCs in the samples from the node-positive patients was greater than that in the node-negative patients (*p* = 0.1752) (Figure 7b). Compared with the patients without distant metastases, those with distant metastases had similar numbers of GFP-positive CTCs (*p* = 0.5655) (Figure 7c). There was not a significant difference between the differentiated and undifferentiated type (*p* = 0.8387) (Figure 7d). The numbers of CTCs were similar in the samples from patients with and without lymphatic invasion (*p* = 0.2054) (Figure 7e). For venous invasion, the number of CTCs in the samples from the patients with invasion was significantly higher than that in patients without invasion (*p* = 0.0351) (Figure 7f).

## Discussion

This paper reports the correlation between CTCs and gastric cancer, which is the second leading cause of cancer-related death worldwide. The usefulness of the detection of CTCs in the diagnosis, estimation of prognosis, and evaluation of treatment effects has already been reported for breast [27,33], prostate [34], lung [35] and digestive tract [11,36,37] cancers. This study indicates that it is also useful for gastric cancer.

One major outcome of our study was a significant relationship between the number of CTCs and prognosis. Although we used a short follow-up period, the prognosis of patients who had ≥5 CTCs in their 7.5-ml peripheral blood samples was poor. Patients with early stage disease generally have a lower recurrence rate [38], and those with stage I disease actually had no recurrence in our study. We also calculated survival rate in advanced gastric cancer patients with stage II–IV disease, and showed that these patients had a similar survival pattern to those with stage I–IV disease. These results suggest that preoperative chemotherapy or comprehensive treatment is appropriate for advanced disease patients who have many CTCs in pretreatment samples because of their poor prognosis.

Among our pathological findings, only venous invasion had a significant relationship with the number of CTCs. This suggests agreement between traditional pathology and modern molecular technology. We believe that the lack of a significant difference between the number of CTCs detected by our assay and the other pathological indices can be explained by methodological issues such as the small sample size. Our technique worked well as a prognostic evaluation tool — to provide information about the need for postoperative adjuvant therapy — and it may provide a useful auxiliary tool to pathology-based classification.

It is not clear whether all detected CTCs have metastatic potential. Viable CTCs were detected in the samples from early-stage cancer patients in the present study, but almost all stage I group patients survived without recurrence [38]. We intend to confirm whether resistant cancer cells, like cancer stem cells, were present in the detected GFP-positive cells. Also, we will analyse the functions of viable CTCs individually after cell sorting, and identify CTCs with metastatic potential using additional tools such as DNA ploidy analysis [39,40]. Furthermore, gene expression profiling among viable CTCs, dead cells, primary tumours, and metastatic tumours will reveal important information related to the mechanisms of cancer metastasis.

Our results must be interpreted with some caution, considering the limitations of this study. First, the number of CTCs varied widely between individuals, including patients and healthy volunteers. Moreover, it was not influenced by tumour stage or the presence of clinically detectable metastases. Similarly, there was no association between the presence of metastases (lymph node or distant) or invasion and the number of CTCs, which brings into question the use of CTCs to predict metastatic potential. However, it is possible that the relatively small sample size limited the potential to detect real differences between these groups of patients. Finally, it must be acknowledged that our results are only applicable to preoperative, treatment-naïve gastric cancer and should not be generalised to other types of cancer.

Clearly, more studies in a larger population of patients, and with different cancer types, are needed to clarify the clinical applicability of CTC detection. We are now preparing a study to investigate the effects of gastric cancer treatment on the number of CTCs, which should help to establish their relationship with future disease progression and whether CTC count can help guidetherapy choice.

## Conclusions

There was a significant difference in survival between patients with 0–4 CTCs and those with ≥5. However, it is unclear whether all CTCs have true metastatic potential, and further studies are needed.

## Competing interests

No competing interests declared.

## Authors’ contributions

HI (Ito) conceived and designed the experiments, collected blood samples, performed the experiments, delivered clinical patients’ data, and performed the statistical analysis and interpretation of data. HI (Inoue) participated in the study design and performed interpretation of data. NS participated in the study design and performed interpretation of data. SK (Kimura) participated in the study design, maintained the equipment, including the test tubes, and performed data interpretation. KG, JS and KM performed the experiments and interpretation of data. SI performed the statistical analysis and interpretation of data. NO and HS managed clinical examination and performed treatment of individual patients. SK (Kudo) participated in the study design and coordination. All authors have read and approved the final manuscript.

## Pre-publication history

The pre-publication history for this paper can be accessed here:

http://www.biomedcentral.com/1471-2407/12/346/prepub
